# The Effect of Propofol vs. Isoflurane Anesthesia on Postoperative Changes in Cerebrospinal Fluid Cytokine Levels: Results from a Randomized Trial

**DOI:** 10.3389/fimmu.2017.01528

**Published:** 2017-11-13

**Authors:** Miles Berger, Vikram Ponnusamy, Nathaniel Greene, Mary Cooter, Jacob W. Nadler, Allan Friedman, David L. McDonagh, Daniel T. Laskowitz, Mark F. Newman, Leslie M. Shaw, David S. Warner, Joseph P. Mathew, Michael L. James, For the MAD-PIA Investigators

**Affiliations:** ^1^Department of Anesthesiology, Duke University Medical Center, Durham, NC, United States; ^2^University of Missouri School of Medicine, Columbia, MO, United States; ^3^Neurosurgical Anesthesiology, Postanesthesia Care Unit, Department of Anesthesiology, University of Rochester, Rochester, NY, United States; ^4^Department of Neurosurgery, Duke University Medical Center, Durham, NC, United States; ^5^Department of Anesthesiology & Pain Management, Neurological Surgery, Neurology and Neurotherapeutics, University of Texas, Southwestern, Dallas, TX, United States; ^6^Department of Neurology, Duke University Medical Center, Durham, NC, United States; ^7^Department of Neurobiology, Duke University Medical Center, Durham, NC, United States; ^8^Private Diagnostic Clinic, Duke University Medical Center, Durham, NC, United States; ^9^Department of Pathology and Laboratory Medicine, Perelman School of Medicine, University of Pennsylvania, Philadelphia, PA, United States; ^10^Department of Surgery, Duke University Medical Center, Durham, NC, United States

**Keywords:** anesthesia, cerebrospinal fluid, cytokine, inflammation, isoflurane, propofol, surgery, neuroinflammation

## Abstract

**Introduction:**

Aside from direct effects on neurotransmission, inhaled and intravenous anesthetics have immunomodulatory properties. *In vitro* and mouse model studies suggest that propofol inhibits, while isoflurane increases, neuroinflammation. If these findings translate to humans, they could be clinically important since neuroinflammation has detrimental effects on neurocognitive function in numerous disease states.

**Materials and methods:**

To examine whether propofol and isoflurane differentially modulate neuroinflammation in humans, cytokines were measured in a secondary analysis of cerebrospinal fluid (CSF) samples from patients prospectively randomized to receive anesthetic maintenance with propofol vs. isoflurane (registered with http://www.clinicaltrials.gov, identifier NCT01640275). We measured CSF levels of EGF, eotaxin, G-CSF, GM-CSF, IFN-α2, IL-1RA, IL-6, IL-7, IL-8, IL-10, IP-10, MCP-1, MIP-1α, MIP-1β, and TNF-α before and 24 h after intracranial surgery in these study patients.

**Results:**

After Bonferroni correction for multiple comparisons, we found significant increases from before to 24 h after surgery in G-CSF, IL-10, IL-1RA, IL-6, IL-8, IP-10, MCP-1, MIP-1α, MIP-1β, and TNF-α. However, we found no difference in cytokine levels at baseline or 24 h after surgery between propofol- (*n* = 19) and isoflurane-treated (*n* = 21) patients (*p* > 0.05 for all comparisons). Increases in CSF IL-6, IL-8, IP-10, and MCP-1 levels directly correlated with each other and with postoperative CSF elevations in tau, a neural injury biomarker. We observed CSF cytokine increases up to 10-fold higher after intracranial surgery than previously reported after other types of surgery.

**Discussion:**

These data clarify the magnitude of neuroinflammation after intracranial surgery, and raise the possibility that a coordinated neuroinflammatory response may play a role in neural injury after surgery.

## Introduction

Many intravenous and inhaled anesthetics modulate immunologic and inflammatory function by acting at multiple receptors and ion channels on leukocytes (1–3). Mouse models and *in vitro* studies have shown that propofol has anti-inflammatory effects (4, 5) while isoflurane has pro-inflammatory effects (6). However, studies comparing the impact of propofol vs. inhaled anesthetics (such as isoflurane) on human postoperative serum inflammatory markers have produced mixed results (7–9). Several studies have also show an increase in cerebrospinal fluid (CSF) cytokine levels after surgery. For example, CSF IL-6 levels increased from before to postoperative day (POD) 1 after hip and knee replacements, aortic valve replacement surgeries, esophageal carcinoma resections, and dural leak repairs (10–14). In 11 patients undergoing endoscopic dural leak repairs, CSF IL-6, IL-10, and TNF-α levels were increased on POD1 and POD2, and sevoflurane-treated patients had a greater increase in CSF IL-6 levels than propofol-treated patients (14). However, anesthetic type was not randomized in this small study, so these results could also be explained by underlying differences between the propofol- vs. sevoflurane-treated patients (14). To the best of our knowledge, no randomized controlled study has compared the impact of different anesthetics or intracranial surgery on postoperative CSF cytokine responses. If anesthetics have inflammatory-modulating properties in humans, this could be clinically significant because many human neurologic and neurocognitive disorders are thought to involve brain inflammation [e.g., multiple sclerosis (MS) (15), traumatic brain injury (TBI) (16), Alzheimer’s disease (AD) (17), human immunodeficiency virus (HIV)-associated neurocognitive dysfunction (18), and postoperative delirium and cognitive dysfunction (19)]. Many patients with these disorders also undergo anesthesia and surgery each year. Thus, understanding the effects of different anesthetics on human postoperative neuroinflammation is an important goal in perioperative medicine.

To evaluate whether isoflurane treatment is associated with a greater postoperative neuroinflammatory response than propofol treatment, we measured CSF cytokine levels before and 24 h after intracranial surgical procedures. CSF samples were obtained from a previously reported trial (20), which was designed to measure changes in CSF AD biomarkers in patients prospectively randomized to receive propofol vs. isoflurane for anesthetic maintenance.

## Patients and Methods

### Patients

After approval by the Duke University Institutional Review Board, intracranial surgery patients whose case posting included lumbar CSF drain placement were enrolled in the Markers of AD after Propofol vs. Isoflurane Anesthesia (MAD-PIA) trial (20). MAD-PIA was a prospective randomized trial registered with http://www.clinicaltrials.gov (NCT01640275) on June 20, 2012 by Miles Berger, the study PI. Patients were excluded if they were (1) unable to provide informed consent, (2) <18 years old, (3) pregnant, (4) imprisoned, or (5) had a personal or family history of malignant hyperthermia or other medical contraindication to receiving propofol or isoflurane. The primary aim of the parent study was to evaluate the effect of anesthetic type on CSF AD biomarkers, such as amyloid beta, tau, and phosphorylated tau (20). For this study, additional CSF sample aliquots collected during the MAD-PIA trial were used to evaluate the effect of anesthetic type on postoperative CSF cytokine increases. Cognitive impairment (clinical AD, mild cognitive impairment, or other cognitive impairment) was not an exclusion criterion, although patients had to be able to give informed consent. We did not exclude intracranial surgical cases with neuromonitoring (such as brainstem auditory evoked responses).

To determine the impact of surgical procedure type on neuroinflammation, surgical cases were divided into three types (Table 2): 1, peripheral neurosurgery without deep intracranial work (e.g., retromastoid craniectomy for trigeminal nerve decompressions, or CSF leak repairs); 2, deep intracranial surgery (e.g., cerebellopontine angle tumor or acoustic neuroma resections); or 3, miscellaneous cases (e.g., cortical surface meningioma resections).

### Study Protocol

Patients were randomized to receive either propofol or isoflurane for anesthetic maintenance; however, all patients received propofol for anesthesia induction. Depending on the patient’s randomization assignment, anesthesiologists (and anesthesia residents and nurse anesthetists) were instructed to titrate propofol or isoflurane dosage to maintain a bispectral index (BIS) value of 40–60. There were no protocol restrictions regarding the use of other anesthetic drugs, such as opioids, paralytics, or steroids. After anesthetic induction but before surgical incision, a member of the surgery team inserted a subdural Silastic^®^ catheter at the L4–L5 or L5–S1 interspace, and then connected it to an external CSF drain (AccuDrain INS-8400; Integra Neurosciences, Plainsboro, NJ, USA).

### CSF Sampling

Fresh CSF samples (10 ml) were obtained from the lumbar drain at the time of drain placement (0 h) and 24 h later, and placed in a 15 ml conical tube (VWR; Radnor, PA, USA) on ice. The CSF samples were divided into 1 ml aliquots in pre-chilled Sarstedt 1.5 ml polypropylene microcentrifuge tubes (VWR; Radnor, PA, USA) with low-binding 1,000 µl pipette tips (Genesee; San Diego, CA, USA) and then stored at −80°C.

### CSF Cytokine Measurement

A wide variety of molecules and cells are involved in various human inflammatory disease states (21–24). Since there were little previously published data on which inflammatory molecules may be involved in the human brain’s response to intracranial surgery, we assessed a broad spectrum of inflammatory markers using the Millipore 15-plex HCYTOMAG-60K plate (Millipore; Billerica, MA, USA), including EGF, eotaxin, G-CSF, GM-CSF, IFN-α2, IL-10, IL-1RA, IL-6, IL-7, IL-8, IP-10, MCP-1, MIP-1α, MIP-1β, and TNF-α. Cytokine assays were performed at the Immune Reconstitution Core Laboratory at the Duke Human Vaccine Institute. CSF samples were diluted 1:40 and then assayed in duplicate using the HCYTOMAG-60K panel. The assay was performed according to the manufacturer’s recommended protocol and data were obtained (to the 100th digit place) using a Bio-Plex 200 array reader (Bio-Rad; Hercules, CA, USA). Data were analyzed using Bio-Plex manager software (Bio-Rad; Hercules, CA, USA).

### Sample Size/Power Analysis

This study aimed to test the hypothesis that propofol and isoflurane have differential effects on postoperative human neuroinflammation. To the best of our knowledge, no prior randomized human study has measured CSF cytokine levels before and after surgery in patients who received propofol vs. isoflurane. Thus, we judged there to be insufficient preliminary data to perform a formal *a priori* sample size calculation. To estimate the potential effect size of anesthetic type upon human postoperative CSF cytokine increases after intracranial surgery, we measured cytokine levels in CSF samples from patients in the MAD-PIA study (20), who were randomized to receive propofol (*n* = 19) vs. isoflurane (*n* = 21) for anesthetic maintenance.

### Statistical Analysis

Baseline and intraoperative differences between the propofol and isoflurane treatment groups were evaluated with *t*-tests for continuous variables and chi-square tests for categorical variables in Table 1. Changes in cytokine levels from before to 24 h after surgery were evaluated with Wilcoxon signed rank tests (Table 3). To account for analysis of multiple cytokines, we used the Bonferroni method to adjust *p* values [Table 3; (25)]. Differences in cytokine levels between isoflurane- vs. propofol-treated patients were compared with Wilcoxon ranked sum tests, at both the baseline and 24 h time points (Figure 2). Spearman correlation coefficients were used to examine the correlation between changes among CSF cytokines over time and between CSF cytokines and CSF tau levels, and a Bonferroni correction for multiple comparisons was performed (Figure 3). A Wilcoxon ranked sum test was used to compare 24 h cytokine levels between patients discharged on POD1 vs. after POD1. For all analyses, significance was set at α = 0.05 after multiple comparison correction. Stata 15 SE (Statacorp, College Station, TX, USA) was used for all statistical analyses.

## Results

**Figure 1:**
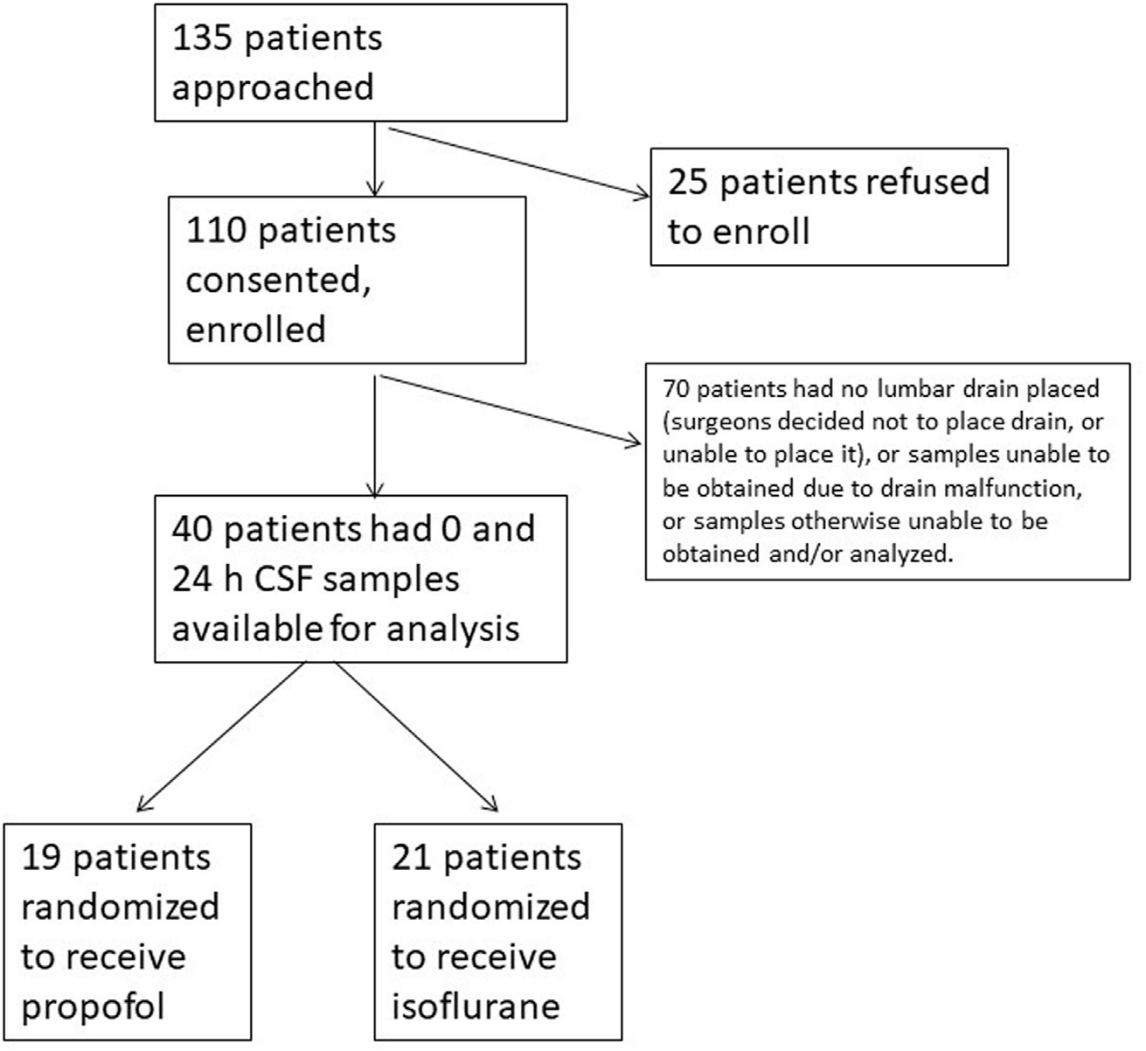
CONSORT diagram/flow chart of the study population. CSF, cerebrospinal fluid.

Study enrollment is depicted in Figure 1. Baseline and intraoperative characteristics of the study patients who had samples obtained at 0 and 24 h are presented in Table 1. None of the patients had a diagnosed neurodegenerative disease or MCI. The isoflurane group was older, received slightly less hydromorphone than the propofol group, and had slightly higher intraoperative bispectral index readings; otherwise, the two groups were generally well balanced. There were more females than males in both groups, but the difference between the groups was not significant. The propofol-treated patients received significantly higher intraoperative propofol dosage, as expected since they were randomized to receive propofol for anesthetic maintenance. There was no significant difference in the surgery type distribution between anesthetic groups (Tables 1 and 2).

**Table 1:** Baseline and intraoperative characteristics for the propofol and isoflurane groups.

	Propofol group (*n* = 19)	Isoflurane group (*n* = 21)	
		
	Mean (SE) or %	Mean (SE) or %	*p*-Value*
**Baseline patient characteristics**			
Age	50.2 (3.4)	59.9 (2.6)	0.034
Sex (male/female)	5.3/94.7%	23.876.2%	0.089
Weight (kg)	79.0 (4.9)	82.3 (3.7)	0.465
Body mass index	29.5 (2.2)	29.0 (1.1)	0.957
**Intraoperative pharmacologic variables**							
Propofol infusion duration (min)	261 (21)	–	
Average propofol drip rate (μg/kg/min)	104.5 (3.7)	–	
Propofol total dosage (mg)	2,114.4 (186.6)	164.1 (27.3)	<0.001
Case length (min)	315 (22)	360 (21)	0.167
Anesthetic duration (min)	311 (22)	361 (23)	0.167
Average MAC^a^	–	0.5 (0)	
Average MAC (h)^a^		2.6 (0.1)	
Total remifentanil dose (mg)	4.2 (0.6)	3.8 (0.5)	0.946
Total fentanyl dose (μg)	258 (20)	252 (11)	0.685
Total hydromorphone dose (mg)	0.9 (0.2)	0.5 (0.1)	0.041
Total dexmedetomidine dose (μg)	7.3 (4.5)	5.6 (1.6)	0.261
Total dexamethasone dose (mg)	13.4 (2.6)	16.9 (2.3)	0.291
Total midazolam dose (mg)	1.4 (0.2)	1.5 (0.2)	0.745
Total clonidine dose (μg)	0 (0)	1.4 (1.4)	0.797
**Intraoperative physiologic variables**							
Heart rate^a^	70 (3)	68 (2)	0.583
Invasive arterial blood pressure^a^	83 (2)	80 (1)	0.085
Temperature^a^	35.8 (0.3)	35.9 (0.2)	0.698
Pulse oximetry^a^	99 (1)	99 (1)	0.692
Bispectral index^a^	41 (2)	47 (7)	0.031
Surgery type^b^			
1	10	9	
2	7	5	0.2153
3	2	7	

*p Values are from Wilcoxon rank sum tests for continuous variables and chi-square test for categorical variables.

*^a^The propofol treatment group was missing data (number of missing patients) for minute to minute heart rate (2), invasive arterial blood pressure (3), case temperature (10), pulse oximetry (2), and bispectral index (11). The isoflurane treatment group was missing data for average minute to minute MAC (2), MAC-hours (2), heart rate (1), invasive arterial blood pressure (2), case temperature (5), pulse oximetry (1), and bispectral index (5). Gross inspection of the q15 minute vital signs from the intraoperative anesthetic records from these cases showed that the missing heart rate, blood pressure, temperature (except for three cases in which temperature was not recorded), and pulse oximetry values were within 1 SD of the means reported above*.

*^b^For surgical type breakdown see Table 2*.

**Table 2:** Surgery types.

Surgery type		Propofol group (*n* = 19)	Isoflurane group (*n* = 21)
1	Cerebrospinal rhinorrhea repair	2	1
	Retromastoid craniectomy	8	8
2	Acoustic neuroma resection	2	1
	Craniectomy to cerebellopontine angle	5	4
3	Craniopharyngioma resection	0	1
	Intracranial hemangioma resection	1	0
	Olfactory groove meningioma resection	0	1
	Meningioma—posterior fossa resection	0	1
	Meningioma—supratentorial resection	1	1
	Orbital lesion	0	1
	Pituitary adenoma resection	0	1
	Trigeminal schwannoma resection	0	1

**Figure 2:**
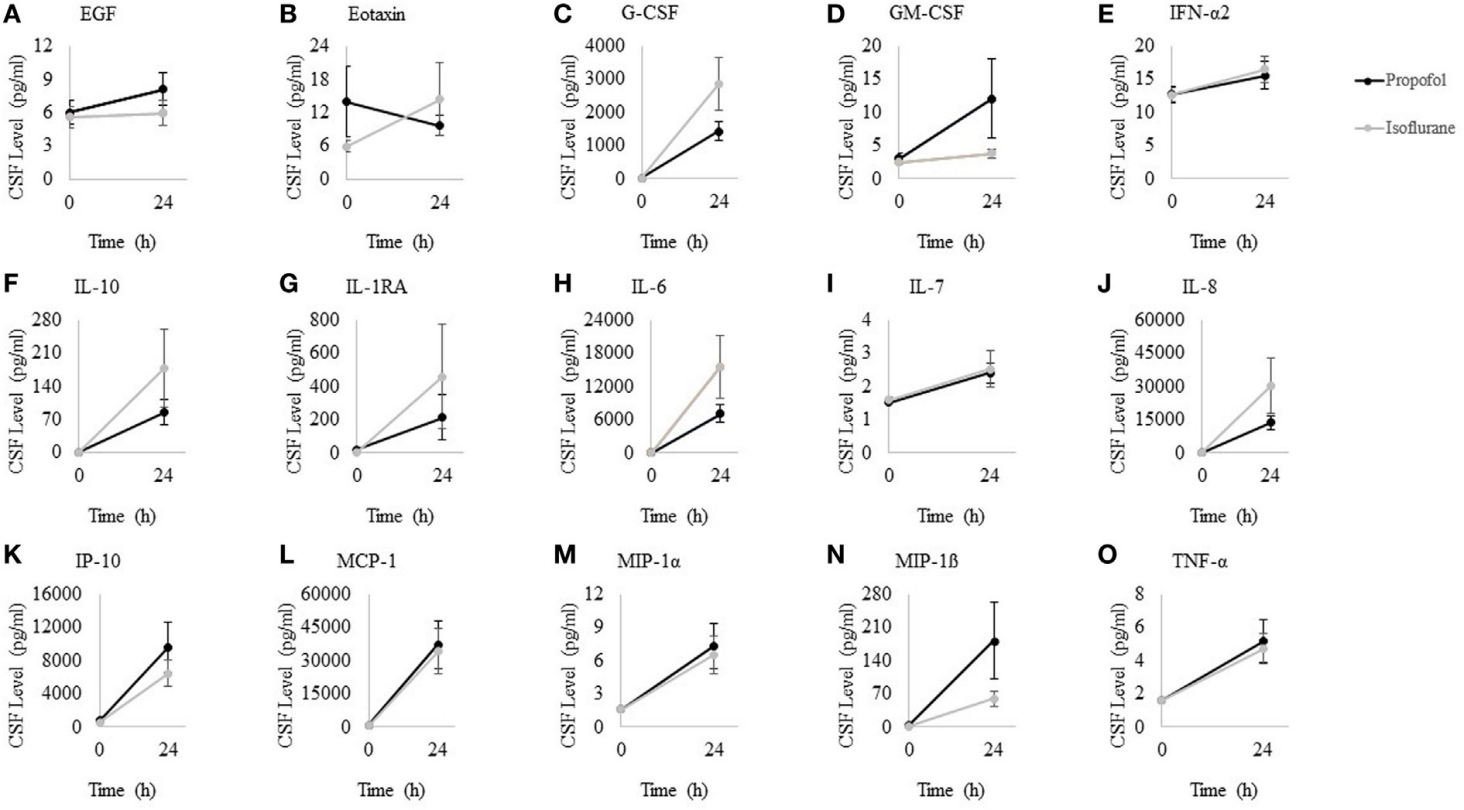
Cerebrospinal fluid (CSF) cytokine levels at 0 and 24 h after surgery for patients randomized to propofol and isoflurane. Wilcoxon rank sum tests showed that was no differences in cytokine levels between propofol and isoflurane-treated patients at either 0 or 24 h (*p* > 0.05 for all, prior to Bonferroni correction). Data points represent anesthetic group means; error bars represent standard error of the mean. **(A)** EGF, **(B)** Eotaxin, **(C)** G-CSF, **(D)** GM-CSF, **(E)** IFN-α2, **(F)** IL-10, **(G)** IL-1RA, **(H)** IL-6, **(I)** IL-7, **(J)** IL-8, **(K)** IP-10, **(L)** MCP-1, **(M)** MIP-1α, **(N)** MIP-1β, and **(O)** TNF-α.

Cytokine levels were measured in CSF samples obtained before and 24 h after surgery. Even after Bonferroni correction (25), G-CSF, IL-10, IL-1RA, IL-6, IL-8, IP-10, MCP-1, MIP-1α, MIP-1β, and TNF-α each showed a significant increase from before to 24 h after surgery (Table 3). However, there were no significant differences between propofol vs. isoflurane-treated patients either before or 24 h after surgery (Figure 2).

**Table 3:** Cerebrospinal fluid cytokine levels before and 24 h after surgery (*n* = 40).

Biomarker	Mean pg/ml (SE) at 0 h	Mean pg/ml (SE) at 24 h	Raw *p* value	Bonferroni corrected *p* value
EGF	5.80 (0.73)	6.98 (0.96)	0.3387	>0.99
Eotaxin	9.70 (3.18)	12.17 (3.59)	0.1344	>0.99
G-CSF	20.77 (6.62)	2,170.23 (455.64)	<0.00005	<0.001
GM-CSF	2.75 (0.43)	7.72 (2.95)	0.0942	>0.99
IFN-α2	12.65 (0.81)	16.03 (1.49)	0.2732	>0.99
IL-10	1.57 (0)	133.31 (45.74)	<0.00005	<0.001
IL-1RA	11.79 (7.26)	342.97 (181.44)	0.0001	0.002
IL-6	1.81 (0.16)	11,543.85 (3149.69)	<0.00005	<0.001
IL-7	1.56 (0.04)	2.47 (0.32)	0.0036	0.054
IL-8	97.35 (22.72)	22,388.83 (6926.77)	<0.00005	<0.001
IP-10	645.57 (112.33)	7,984.34 (1,718.99)	<0.00005	<0.001
MCP-1	766.13 (120.82)	35,689.67 (7,469.17)	<0.00005	<0.001
MIP-1α	1.61 (0)	6.92 (1.33)	<0.00005	<0.001
MIP-1β	2.76 (0.6)	117.68 (40.85)	<0.00005	<0.001
TNF-α	1.60 (0)	4.92 (0.78)	<0.00005	<0.001

**Figure 3:**
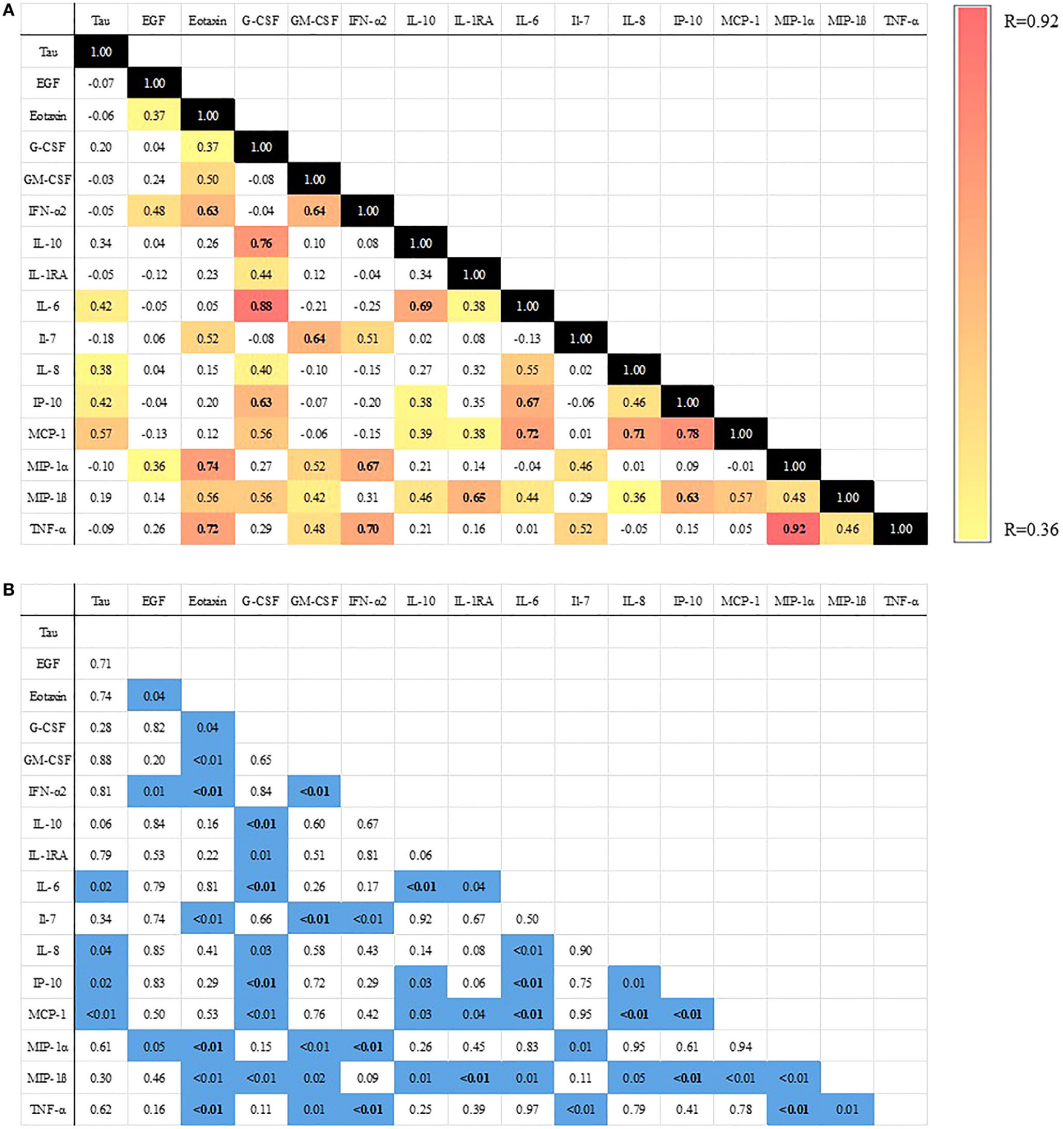
Heat map of Spearman correlations between change in cerebrospinal fluid (CSF) cytokine levels from 0 to 24 h after surgery. **(A)** All colored cells are significant at *p* < 0.05. Bolded cells are significant after Bonferroni correction (25). Color bar ranges from yellow (*R* = 0.36 or smallest significant spearman correlation) to red (*R* = 0.92 or largest significant spearman correlation). **(B)** *p* Values for each comparison in part A [values <0.05 highlighted in blue and values significant after Bonferroni correction are bolded (25)].

To determine whether postoperative changes in CSF cytokines were correlated with one another, we calculated spearman correlation coefficients between each two-way cytokine combination (i.e., 120 possible two-way combinations). Roughly 40% (or 46) of these 120 possible two-way cytokine elevations were positively correlated with one another (Figure 3). Of these correlations, 18 remained significant after Bonferroni correction (25), consistent with a coordinated neuroinflammatory response. We previously reported a trend toward an inverse correlation between intraoperative dexamethasone dosage and the postoperative increase in the neural injury biomarker tau (20), raising the possibility that a steroid-sensitive inflammatory mechanism may be involved in postoperative CSF tau increases. Thus, we examined whether CSF cytokine increases correlated with postoperative CSF tau increases (20). Postoperative increases in CSF IL-6, IL-8, IP-10, and MCP-1 levels each showed a statistically significant correlation with the postoperative increase in CSF tau levels (Figure 3).

Since neuroinflammation is thought to have detrimental effects on cognition and might thus be expected to be associated with prolonged hospital length of stay, we also examined the relationship between postoperative cytokine increase and discharge date. Eight of the 40 total patients were discharged by POD 1. There was no difference in CSF cytokine levels from before to 24 h after surgery in the 8 patients discharged at POD 1 vs. the 32 patients discharged after POD 1 [*p* > 0.05 for each cytokine, after Bonferroni correction (25)].

## Discussion

Here, we report that anesthetic type had no significant effect upon CSF cytokine levels after intracranial surgery, yet multiple CSF cytokine levels increased significantly after surgery, and many of these CSF cytokine increases correlated with one another and with CSF tau increases. Our comparison of propofol- and isoflurane-treated patients was limited by slight differences in age, hydromorphone dose, and BIS index (Table 1), though we believe that these slight differences between groups are unlikely to have obscured a biologically significant difference in cytokines levels. Our results are similar to those of Beck-Schimmer et al., who recently found no difference in LPS-induced neuroinflammation among rats treated with propofol vs. the inhaled anesthetic sevoflurane (26). However, due to insufficient prior literature comparing the human postoperative increase in CSF cytokines between propofol- vs. isoflurane-treated patients, we were unable to perform an *a priori* sample size calculation for this study. Thus, we cannot exclude the possibility that propofol and isoflurane have differential effects on human postoperative neuroinflammation, which may not have been detected here due to a type II statistical error. Nonetheless, the data presented here allow us to perform a *post hoc* sample size calculation for future studies designed to assess whether these anesthetics have differential effects on postoperative CSF cytokine increases. To have 80% power with an α = 0.05 to detect a difference similar in magnitude to that seen here for 24 postoperative vs. baseline CSF cytokine levels in propofol- and isoflurane-treated patients, a future study would need the following sample sizes for each cytokine: 126 for G-CSF; 178 for IL-6; 208 for IL-8; 436 for IP-10; 8,450 for MCP-1; 3,886 for MIP-1α; and 3,422 for TNF-α.

Neuroinflammatory responses in other neurocognitive diseases typically involve changes in the levels of numerous cells and molecules over varying time courses (22–24). Of these, here we measured 15 CSF cytokines; future studies should examine other important cytokines, such as IFN-γ and IL-17. The CSF samples in this study were obtained from patients who had CSF drains. CSF drain placement itself may cause an inflammatory response (27), but we found that much higher CSF cytokine increases than other studies that obtained CSF from an intrathecal drain (11, 14), suggesting that CSF drain placement was not the primary cause of the cytokine increases seen here. Indeed, compared to past studies analyzing CSF cytokines after orthopedic, cardiac, gastrointestinal, and CSF leak repair procedures, this study in intracranial surgery patients found approximately a 65× higher level of MCP-1 (11), a 40× higher level of IL-8 (11–13), and a 25× higher level of IL-6 (10, 11, 13, 14) 1 day after surgery. This comparison is limited by the fact that the patients in these prior studies may have differed from the patients in this study in other baseline characteristics (aside from surgery type). Nonetheless, the massive increases in CSF cytokines reported here raise the possibility that intracranial surgical procedures may be associated with a greater neuroinflammatory response than other types of surgery.

We also found significant correlations among many of these CSF cytokine increases (Figure 3), consistent with a coordinated postoperative neuroinflammatory response. Also, CSF IL-6, IL-8, IP-10, and MCP-1 increases correlated with postoperative increases in the CSF neural injury marker tau (Figure 3). Pikwer and colleagues recently reported similar though smaller increases in many of these same cytokines in the CSF within 7 h after hysterectomy cases, although they found no tau increase and no correlation between cytokine increases and tau levels (28). The lack of correlation between cytokine increases and tau levels in the Pikwer et al.’s study likely reflects the shorter time course in that study (7 vs. 24 h here), since many of the cytokine trajectories in that paper were still increasing at 7 h, as well as the potentially greater impact of neurosurgery upon the brain (28).

Nonetheless, similar correlations between CSF cytokines and neural injury markers (as well as neurocognitive decline) as those seen here have been reported in both AD (17, 29) and TBI (16). In TBI patients, CSF IL-6 levels of ~10,000 pg/ml have been measured 1 day after trauma (30), and here we found CSF IL-6 levels of ~10,000 pg/ml 24 h after surgery (Table 3). Interestingly, CSF IL-10 levels in survivors and non-survivors of TBI at 24 h after trauma were <10 pg/ml (31), while we found IL-10 levels of ~100 pg/ml 24 h after surgery. This further underscores the magnitude of the neuroinflammatory response after intracranial surgery. Taken together, these findings raise the possibility that neuroinflammation after intracranial surgery may contribute to postoperative CSF tau elevation in a similar fashion as that seen in AD and TBI, potentially contributing to neuronal dysfunction after intracranial surgery. Alternatively, CSF cytokine increases may occur secondary to or in response to CSF tau increases (a marker of neuronal damage); future studies will be required to determine the causal relationship between postoperative CSF cytokine and tau increases.

Irrespective of whether CSF cytokine increases cause tau increases or *vice versa*, prior studies have clearly shown that neuroinflammation impairs neurocognitive function at multiple levels ranging from synaptic transmission (32) to functional brain network connectivity (33) to human cognitive performance (34). For example, IL-8 inhibited hippocampal long-term potentiation *in vitro* (32). In addition, intravenous LPS treatment in healthy adults caused altered resting-state functional connectivity within multiple human brain networks (33) and caused increases in IL-6, IL-10, and TNF-α levels in serum and in IL-6 levels within the CSF (35). In fact, neuroinflammation is thought to contribute to both postoperative cognitive dysfunction (POCD) and delirium in older adults (19). Indeed, in a prior study, patients with delirium had higher CSF IL-8 levels (~70 pg/ml) than those without delirium (~40 pg/ml) (36). Furthermore, neuroinflammation has been associated with impaired cognitive performance in multiple human diseases ranging from MS (15) to TBI (16) to AD (17) and HIV (18). However, the role and functional sequelae of the postoperative CSF cytokine increases measured here are unclear. The patients in this trial were not screened for POCD or delirium, as these measures were beyond the scope of this study. Nonetheless, many of these study patients had grossly normal cognition on POD1, and eight were discharged home on POD1. In addition, the discharge summary of 39 of the 40 patients did not contain any indication of delirium, altered mental status, or agitation, although this may reflect a lack of sensitivity in delirium detection/documentation in routine clinical care. There was also no difference in postoperative CSF cytokine increases between the eight patients who were discharged POD1 and those who were discharged after POD1. Together, these results raise the possibility that neuroinflammation alone may be insufficient to cause POCD or delirium, though it may play a role in POCD or delirium in concert with other age-dependent brain changes (37). The magnitude of CSF cytokine increases measured here, together with the known role of neuroinflammation in cognitive impairments in multiple other clinical settings (15–19), suggest that future studies are warranted to determine the cognitive and functional sequelae of postoperative neuroinflammation.

In conclusion, while we found no evidence of an effect of anesthetic on postoperative CSF cytokine increases, there was a highly significant postoperative increase in multiple cytokines. Many of these cytokine increases (i.e., IL-6, IL-8, IP-10, and MCP-1) directly correlated with one another and with CSF tau increases, consistent with a coordinated postoperative neuroinflammatory response. Future studies are needed to further characterize this postoperative neuroinflammatory response and its clinical implications.

## Ethics Statement

This study was carried out with approval from and in accordance with the regulations of the Duke University Medical Center Institutional Review Board. All subjects gave written informed consent in accordance with the Declaration of Helsinki, before any study activities began. The protocol was approved by the Duke University Medical Center Institutional Review Board.

## Author Contributions

MB conceived of, designed, and oversaw this study, and organized the manuscript. VP and MB wrote the manuscript. JN, MB, AF, DM, DW, and MJ helped enroll patients and obtain samples. MC helped in data extraction and design of statistical analysis, NG helped in design of and performed statistical analysis. LS helped perform and oversee assay measurements. JM, MN, DM, and MJ helped design and oversee the study. All authors made substantial contributions to this work, helped revise the manuscript, approved the final manuscript, and agreed to be held accountable for all aspects of the work in ensuring that questions related to the accuracy or integrity of any part of the work are appropriately investigated and resolved.

## Conflict of Interest Statement

MB acknowledges research funding from Minnetronix, Inc., for a study unrelated to the topic of this manuscript, material support from Massimo for another study unrelated to the topic of this manuscript, and private consulting income for a legal case related to postoperative cognition in an older adult. The rest of the authors have no other conflicts to report. The reviewer RL declared a shared affiliation, with no collaboration, with one of the authors, LS, to the handling editor.
